# Theoretical Research on Thermal Shock Resistance of Ultra-High Temperature Ceramics Focusing on the Adjustment of Stress Reduction Factor

**DOI:** 10.3390/ma6020551

**Published:** 2013-02-18

**Authors:** Dengjian Li, Weiguo Li, Dingyu Li, Yushan Shi, Daining Fang

**Affiliations:** 1 State Key Laboratory of Coal Mine Disaster Dynamics and Control, College of Resources and Environmental Science, Chongqing University, Chongqing 400030, China; E-Mails: cwldj@cqu.edu.cn (De.L.); lidingyu@cqu.edu.cn (Di.L.); ysshi@cqu.edu.cn (Y.S.); 2 State Key Laboratory for Turbulence and Complex Systems, College of Engineering, Peking University, Beijing 100871, China; E-Mail: fangdn@pku.edu.cn

**Keywords:** ultra-high temperature ceramics, stress reduction factor, the second thermal shock resistance parameter, constraint

## Abstract

The thermal shock resistance of ceramics depends on not only the mechanical and thermal properties of materials, but also the external constraint and thermal condition. So, in order to study the actual situation in its service process, a temperature-dependent thermal shock resistance model for ultra-high temperature ceramics considering the effects of the thermal environment and external constraint was established based on the existing theory. The present work mainly focused on the adjustment of the stress reduction factor according to different thermal shock situations. The influences of external constraint on both critical rupture temperature difference and the second thermal shock resistance parameter in either case of rapid heating or cooling conditions had been studied based on this model. The results show the necessity of adjustment of the stress reduction factor in different thermal shock situations and the limitations of the applicable range of the second thermal shock resistance parameter. Furthermore, the model was validated by the finite element method.

## 1. Introduction

Ultra-high temperature ceramics (UHTCs) are a family of ceramic-based composites mainly consisting of transition metal compounds, such as ZrB_2_, TaC, HfN and HfB_2_, which have melting points higher than 3,000 °C and can be potentially used at temperatures above 2,000 °C in an oxidizing environment. As the most promising candidates for high temperature applications of thermal protection systems (TPS), UHTCs are attracting more and more attention currently [1,2,3,4].

The thermal shock resistance (TSR) is one of the most important parameters in UHTCs’ characterizations, since it determines their performances in many applications. Due to their inherent brittleness and poor TSR performance, catastrophic failure may occur under severe thermal shock, which is one of the most important reasons for ceramic fracture [5]. Therefore, improving the TSR of ceramics has been one of the most important focal points in the ceramics field.

Significant progress has been made in the understanding of the thermal shock behavior of ceramic materials, with great efforts of theories and experiments since the 1950s [5,6,7,8,9]. Theoretical research mainly focused on the factors that affect the TSR of ceramics by simplifying the models of the thermal stress field and the transient temperature field. Thus, the stress reduction factor was introduced in order to simplify the analysis process [5,6,7]. At present, the research of TSR mostly focused on the effects of surface defects, temperature, indentation crack length [10,11], particle reinforced [12], whisker reinforced [13] or initial stress field [14] on TSR performance to explain the mechanisms of thermal shock failure. However, few experiments have considered the influences of external constraint conditions, because they are difficult to induct.

It is known to all that the UHTCs are always a part of the TPS; it must be constrained by other parts. So, the TSR performance of the UHTCs is sensitive to the constraint. Thus, the TSR of the material cannot be simply considered on its own, but needs to take the external constraint conditions and the thermal environment into full account. However, in the current experiment it is difficult to simulate the thermal environment and external constraint conditions suffered by the UHTCs, which were used as thermal protection materials.

Due to the restrictions of current experiments, in the present investigation, a TSR model considering the effects of the thermal environment and external constraint had been established. The adjustment of stress reduction factor was considered and the influences of external constraint on both critical rupture temperature difference and the second TSR parameter in either case of rapid heating or cooling conditions had been studied. The present work was limited to establishing the TSR theoretical model and its validation by finite element simulation, which the experimental validation deferred to for future work.

## 2. Derivation of the Theoretical Model

**Figure 1:**
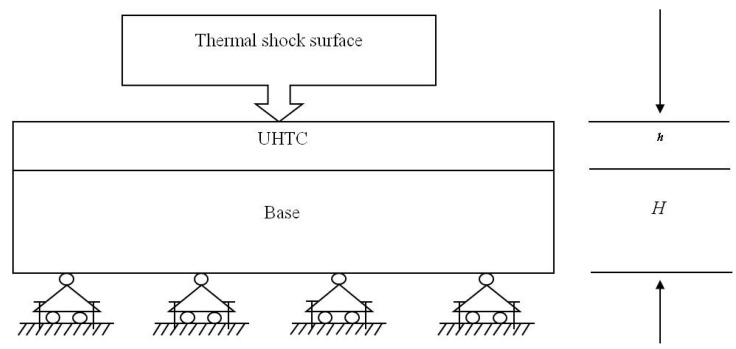
The geometric model.

The geometric model is shown in Figure 1. Assumptions that have been adopted are given below.

1. The model is a two well-bonded plate, which doesn’t consider the interface damage.
2. The upper is the UHTC plate, and the lower is the matrix base. These two plates are assumed to have the same plane geometry size for the convenience of theoretical model derivation.
3. There is no heat exchange between the UHTC plate and the matrix base, and the temperature of the matrix base is constant, being equal to the predefined room temperature field of 25 °C.
4. The plate is continuous, homogenous, isotropic, elastic and submits to small deformation hypothesis.

So, the stress field of the ceramic layer is only the function of thickness direction when it suffers thermal shock. Once the thermal stress is greater than the fracture strength of the ceramics, cracks will be generated and result in instant fracture [5,7]. Besides, the maximum stress appears at the surface for most cases, so it is very reasonable to regard the UHTC plate rupture once the thermal stress of the upper surface caused by thermal shock is greater than the fracture strength of the material corresponding to the current temperature.

### 2.1. Heating Thermal Shock Conditions

The initial stress field is set up by slow heating from the predefined room temperature of 25 °C to the thermal shock initial temperature *T* uniformly without an internal temperature gradient [14]. Then, the model is subjected to a heating thermal shock.

If the temperature of the ceramic plate changes without external restriction, the elongation is ∆*L*_1_. Considering the constraint of the base plate, the expansion of the ceramic plate will be restricted, pressure stress σ will occur in the ceramic and the elongation of the plate caused by the pressure stress will be ∆*L*_σ_. So, the total elongation of the ceramic plate ∆*L* should be the sum of both:

(1) $$\Delta L=\Delta L1+\Delta L\sigma =\left[\alpha \left(T-T_{i}\right)-\frac{\sigma }{E_{c}}\left(1-\nu_{c}\right)\right]L$$

where both the length and width of the ceramic plate are equal to *L.* *α* is the thermal expansion coefficient, and *T_i_* is the predefined room temperature of 25 °C.

Known from the third Newtonian law, a tensile stress will be produced in the base material, the magnitude of which is also σ. Then, the elongation of the matrix under the tensile will be ∆*L*’. By the assumption that the two plates are well-bonded and not bending, ∆*L* and ∆*L*’ are equal, so σ can be derived as follows:

(2) $$\sigma =\frac{E_{\text{B}}E_{c}\alpha \left(T-T_{i}\right)}{E_{B}\left(1-\nu_{c}\right)+E_{c}\left(1-\nu_{\text{B}}\right)}$$

When considering the effects of temperature on the UHTC’s material properties, the formula will be:

(3) $$\sigma =\frac{E_{\text{B}}E_{c}\left(T\right)\alpha \left(T\right)\cdot \left(T-T_{i}\right)}{E_{B}\left(1-\nu_{c}\right)+E_{c}\left(T\right)\cdot \left(1-\nu_{\text{B}}\right)}$$

where *E_B_* and *v_B_* are Young’s modulus and the Poisson ratio of the base plate, respectively. *E_c_*(*T*) and *α*(*T*) are Young’s modulus and the thermal expansion coefficient of the ceramic material at temperature *T*, respectively.

The relationship between Young’s modulus and temperature is assumed to satisfy the following relation [15]:

(4) $$E=E_{0}-B_{0}Te^{-\frac{T_{m}}{T}}+B_{1}(T-B_{2}T_{m}+|T-B_{2}T_{m}|)e^{-\frac{T_{m}}{T}}$$

where *E*_0_ is Young’s modulus at 0 °C, *T*_m_ is the melting point and *B*_0_, *B*_1_, *B*_2_ are material constants.

The temperature dependent strength σ_f_(*T*) of the UHTCs is shown in Equation (5) [16]:

(5) σf(T)=[(σth0)2E0E(T)[1−1∫0TmCp(T)dT∫0TCp(T)dT]]12

where $\sigma_{th}^{0}$ is the fracture strength at the reference temperature, *E*_0_ is Young’s modulus at the reference temperature of the material and *E*(*T*) is the temperature-dependent Young’s modulus. *C_p_*(*T*) is the specific heat capacity for constant pressure, and *T*_m_ is the melting point of material.

Combining the Equation (3) and the second TSR parameter, *R’*, considering only the effect of the thermal environment from reference [17], it is easy to know that the second TSR parameter, *R’*, considering the effects of the thermal environment and the external constraint, and can be solved out as Equation (6):

(6) $$R^{\prime }=\left[\sigma_{f}\left(T+\Delta T_{c}\right)-\frac{E_{\text{B}}E_{\text{c}}\left(T\right)\alpha \left(T\right)\cdot \left(T-T_{i}\right)}{E_{B}\left(1-\nu_{\text{c}}\right)+E_{\text{c}}\left(T\right)\cdot \left(1-\nu_{\text{B}}\right)}\right]\cdot \frac{k\left(T+\Delta T_{\text{c}}\right)\left(1-\nu_{\text{c}}\right)}{E_{\text{c}}\left(T+\Delta T_{\text{c}}\right)\alpha \left(T+\Delta T_{\text{c}}\right)}$$

where σ_f_(*T* *+* ∆*T_c_*) and *k*(*T* *+* ∆*T_c_*) are the temperature-dependent fracture strength and thermal conductivity at temperature* T + ∆T_c_*, respectively.

Generally, the stress reduction factor is used to analyze the thermal stress so as to study the TSR of the materials [5,6,18]. To obtain the critical rupture temperature difference, the stress reduction factor needs to be evaluated using the Biot number and dimensionless time.

For an infinite ceramic plate after a sudden temperature change ∆*T*, the stress reduction factor of the surface can be expressed as follows [5,6,18]:

(7) $$\phi =\frac{\sigma (t)}{{\left(1-\nu \right)}^{-1}\alpha E\Delta T}$$

where *ϕ* is the stress reduction factor and σ(*t*) is the actual thermal stress field of the plate surface at time* t*. The expression of the numerator, σ(*t*), contains the Biot number, *β*, and dimensionless time, *F*_0_, which are used in the evaluation of stress reduction factor [19]. The denominator represents the possible maximum stress when the temperature of the surface is instantly changed to the external temperature, while the other regions remain unchanged.

The Biot number,* β*, is defined as follows [18]:

(8) $$\beta =\frac{hts}{k}$$

where *h* is the thickness of ceramic plate, *t_s_* is the surface heat transfer coefficient and *k* is thermal conductivity.

The dimensionless time is defined as [18,20]:

(9) $$F_{0}=\frac{kt}{C_{P}\rho h^{2}}$$

where *C_p_* is the specific heat capacity for constant pressure, *ρ* is density and *t* is time.

Normally, when the temperature change was relatively slow, Manson found that the value of the stress reduction factor is 0.31*β* [20], and later, this conclusion was widely cited [5,6,18]. So, the critical rupture temperature difference is as follows [18,20]:

(10) $$\Delta T_{c}=\frac{R\prime }{0.31hts}.$$

However, it can be seen from its definition formula [Equation (7)] that the stress reduction factor represents the ratio of the actual thermal stress and the possible maximum stress of the surface. So, parameter *ϕ* ranges from 0 to 1. When the value of the Biot number is larger, the temperature changes infinitely fast, *ϕ* is equal to 1, and it decreases as the temperature change slows down. So, depending on different thermal shock processes, the value of the stress reduction factor needs to be adjusted.

As it is subject to the combined effect of the Biot number and dimensionless time, we can assume that:

(11) ϕ=Cβ

where *C* is a constant, which reflects the impact of the Biot number and dimensionless time, corresponding to different thermal shock processes.

Based on Equation (10), the critical rupture temperature difference ∆*T_c_* corresponding to *R’* can be calculated using the following equation:

(12) $$\Delta T_{c}=\frac{R\prime }{Chts}$$

The combination of Equations (6) and (12) yields:

(13) $$\Delta T_{\text{c}}=\frac{1}{Cht_{s}}\left[\sigma_{f}\left(T+\Delta T_{\text{c}}\right)-\frac{E_{\text{B}}E_{\text{c}}\left(T\right)\alpha \left(T\right)\cdot \left(T-T_{i}\right)}{E_{B}\left(1-\nu_{\text{c}}\right)+E_{\text{c}}\left(T\right)\cdot \left(1-\nu_{\text{B}}\right)}\right]\cdot \frac{k\left(T+\Delta T_{\text{c}}\right)\left(1-\nu_{\text{c}}\right)}{E_{\text{c}}\left(T+\Delta T_{\text{c}}\right)\alpha \left(T+\Delta T_{\text{c}}\right)}$$

### 2.2. Cooling Thermal Shock Conditions

For a cooling process, we set the temperature of an UHTC plate heating up from the predefined room temperature of 25 °C to the thermal shock initial temperature *T* uniformly [14]. Then, the model is subjected to a cooling thermal shock

The second TSR parameter,* R’*, can be solved by Equation (14):

(14) $$R^{\prime }=\left[\sigma_{\text{f}}\left(T-\Delta T_{c}\right)+\frac{E_{B}E_{c}\left(T\right)\alpha \left(T\right)\cdot \left(T-T_{i}\right)}{E_{B}\left(1-\nu_{c}\right)+E_{c}\left(T\right)\left(1-\nu_{B}\right)\cdot }\right]\times \frac{k\left(T-\Delta T_{c}\right)\left(1-\nu_{c}\right)}{E_{c}\left(T-\Delta T_{c}\right)\alpha \left(T-\Delta T_{c}\right)}$$

The critical rupture temperature difference, ∆*T_c_*, can be solved as follows:

(15) $$\Delta T_{c}=\frac{1}{Cht_{s}}\left[\sigma_{\text{f}}\left(T-\Delta T_{c}\right)+\frac{E_{B}E_{c}\left(T\right)\alpha \left(T\right)\cdot \left(T-T_{i}\right)}{E_{B}\left(1-\nu_{c}\right)+E_{c}\left(T\right)\left(1-\nu_{B}\right)\cdot }\right]\times \frac{k\left(T-\Delta T_{c}\right)\left(1-\nu_{c}\right)}{E_{c}\left(T-\Delta T_{c}\right)\alpha \left(T-\Delta T_{c}\right)}$$

### 2.3. Finite Element Model

**Figure 2:**
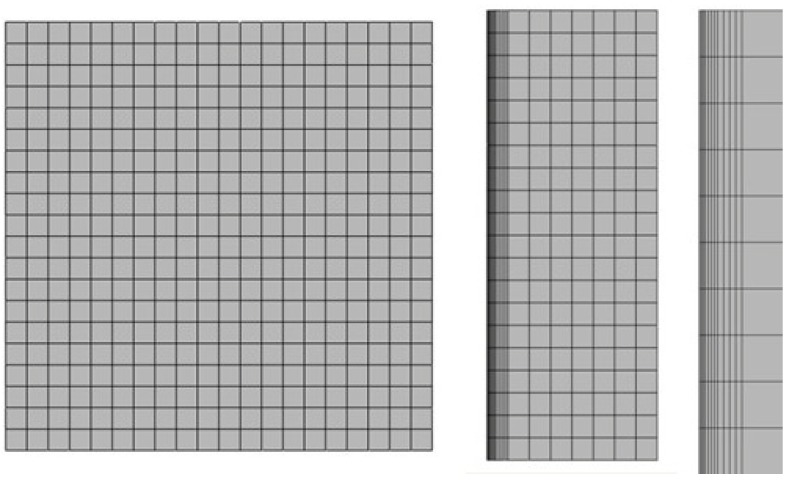
The top view, right view and partial enlarged view of the computational mesh.

Due to the lack of experimental data, the finite element method was used to validate the theoretical model. The numerical simulation was accomplished by using the software SIMULIA Abaqus 6.10-1. According to the symmetry of the model, one-fourth of the plate is used in the numerical simulation. The computational mesh is shown in Figure 2 (length and width are equal to 150 mm; the thickness of matrix base is 50 mm, and the thickness of UHTC plate is 7 mm). The C3D20RT element is used for the UHTC plate and the C3D8T element for the matrix base. The left and lower surfaces are restricted by applying the symmetric constraint, and the displacement of matrix base in the thickness direction is zero.

## 3. Results and Discussion

The relative parameters were obtained from experiments [3,21] or extrapolated from known values at other temperatures, as shown in Table 1. The Poisson ratio of the matrix base was equal to the UHTC plate, and the surface heat transfer coefficient was fixed in the calculation. The thermal shock behavior of HfB_2_ was calculated and analyzed by using the TSR parameter expression, considering the effects of the thermal environment and the external constraint above.

**Table 1:** Temperature-dependent material properties of HfB_2_ [3,21].

Material parameter	Values and expressions
*E*(*T*) (GPa)	See Equation (4)
*E*_0_ (GPa)	440.733
*B*_0_, *B*_1_, *B*_2_	2.54, 1.9, 0.363
$\sigma_{th}^{0}$ (MPa)	448
ν	0.12
*T*_m_ (°C)	3400
*C*_p_(*T*) [J/(kg·°C)]	1532.8 + 1.635 × 10^−^^1^ × (*T +* 273.15) − 4.8086 × 10^7 ^× (*T +* 273.15)^−2^
*k* [W/(m·°C)]	−8.3455 × ln*T* + 127.68
Α (°C^−1^)	(2ln*T* − 5) × 10^−6^

If the value of the Biot number and dimensionless time were determined, we could easily get the value of the stress reduction factor [19]. However, when taking into account the effects of temperature on the material properties, both the Biot number and dimensionless time are functions of temperature, which change continuously in the entire thermal shock process:

(16) $$\beta =\frac{hts}{k(T)};F_{0}=\frac{k(T)t}{C_{P}(T)\rho h^{2}}$$

So, we can’t obtain the accurate value of constant *C* during each specific process. However, at the same time, we found that the range of *C* is extremely small by calculation. For instance, in view of the cooling thermal shock process of which the initial thermal shock temperature was 1,300 °C, we calculated the value of *C* in the condition of* T* (max), *T* (min) and *T* (avg), respectively. As shown in Table 2, its value changes in the range of ±1%. According to Equation (12), the value of the critical rupture temperature difference ∆*T_c_* is directly proportional to 1/C, so only extremely small changes occur in the calculated values of ∆*T_c_*.

So, the temperature dependence of the Biot number and dimensionless time can be negligible in the calculation of the follow-up questions, because the range of the critical rupture temperature difference changes in small scope.

**Figure 3:**
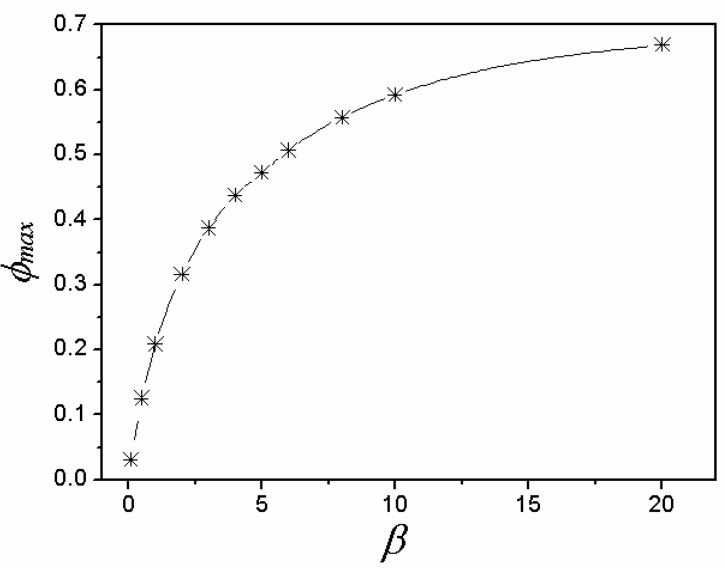
Relationship between the Biot number, *β*, and the maximum value of stress reduction factor, *φ*_max_.

In the actual process of thermal shock, with the increase of dimensionless time, *F*_0_, stress reduction factor *φ* increases rapidly and then decreases slowly, corresponding to the determined Biot number, *β*. Besides, fracture occurs at a time when *φ* approaches its maximum [18,19,20]. Because the range of *F*_0_ is extremely small in this process, a curve, which shows the relationship between *φ*_max_ and *β*, is fitted according to the relationship of the stress reduction factor, the Biot number and dimensionless time (Figure 3). Also, as shown in Table 3, the corresponding value of coefficient *C* in the case of different plate thicknesses is concluded and summarized, based on Figure 3.

**Table 2:** The related material parameters of HfB_2_ in the condition of *T* (max), *T* (min) and *T* (avg), respectively (the cooling rate is assumed to be 200 °C·s^−1^, and the initial temperature is 1,300 °C).

Material parameters	Values	Values	Values
*T* (°C)	1300	965	1132.5
*h* (m)	0.007	0.007	0.007
ts[W /(m^2^·°C)]	2 × 10^4^	2 × 10^4^	2 × 10^4^
ρ (kg/m^3^)	1.05 × 10^4^	1.05 × 10^4^	1.05 × 10^4^
*k* [W/(m·°C)]	67.84	70.33	68.99
*C*_p_(*T*) [J/(kg·°C)]**	1.771 × 10^3^	1.704 × 10^3^	1.738 × 10^3^
β	2.06	1.99	2.03
*F*_0_	0.125	0.134	0.129
C	0.1551	0.1583	0.1565

**Table 3:** Coefficient *C* [18,19].

Plate thickness: *h*(m)	*C*
0.007	0.16
0.014	0.11
0.021	0.084
0.028	0.07
0.035	0.06
0.042	0.05

### 3.1. Adjustment of Stress Reduction Factor

**Figure 4:**
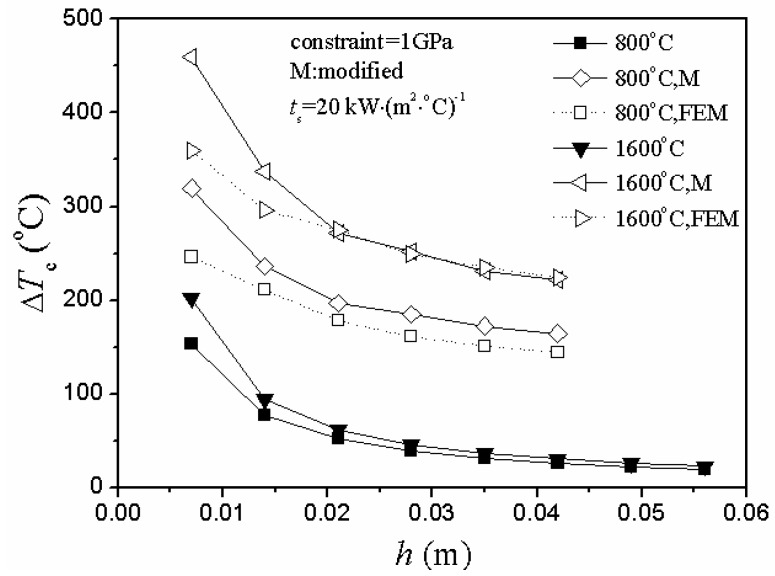
Relationship between critical rupture temperature difference, ∆*T_c_*, and plate thickness, *h*, including modified, unmodified and numerical simulation situations under different initial thermal shock temperature of temperature elevated conditions (FEM represents finite element method).

**Figure 5:**
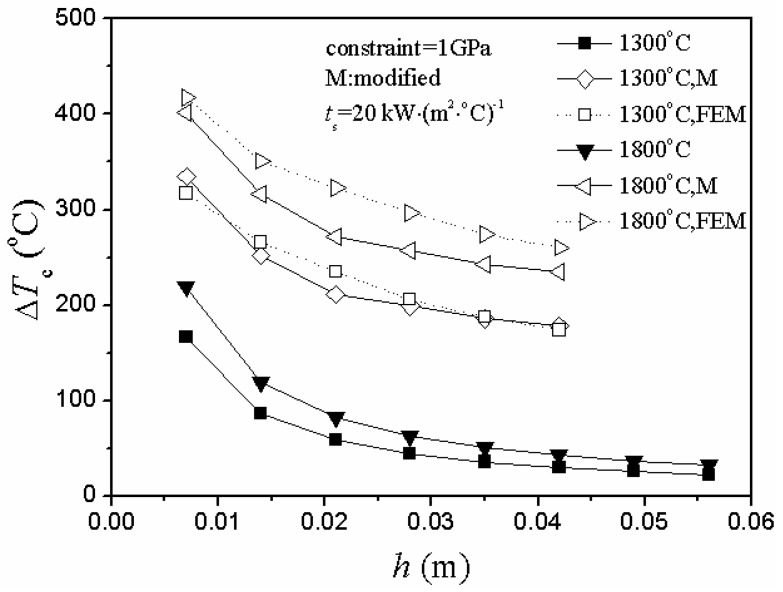
Relationship between critical rupture temperature difference, ∆*T*_c_, and plate thickness, *h*, including modified, unmodified and numerical simulation situations under different initial thermal shock temperature of cooling conditions (FEM represents finite element method).

In either case of heating or cooling conditions, there were two obvious unreasonable aspects of the critical rupture temperature difference represented by the curve of the unmodified situation in Figure 4 and Figure 5.

1. As the plate thickness increased, the critical rupture temperature difference gradually decreased and slowly approached zero.
2. There was a big difference between the theoretical value and the numerical simulation value of the critical rupture temperature difference in the unmodified situation.

However, the adjustment of the stress reduction factor led to the optimized situation:

1. The theoretical and simulation results shared the same trend in different thermal shock conditions, and the value range of the modified situation was more similar to the simulation value.
2. As the plate thickness increased, the theoretical value of the critical rupture temperature difference gradually decreased and slowly approached a constant (nonzero). Besides, the difference between the theoretical and simulation results also gradually decreased, and the entire control results tended toward convergence.

Generally, for problems under the conditions of convection and radiation, the critical rupture temperature difference is negatively correlated with the thickness [5,20,22,23,24,25,26]. Thus, it is quite reasonable that the values of the critical rupture temperature difference decrease with the increase of plate thickness.

Normally, when the temperature change was relatively slow, Manson found that the value of the stress reduction factor is 0.31*β* [20]. However, it is not applicable to all cases. It would bring large deviations and errors when not used in accordance with the thermal environment. So, it is absolutely necessary to adjust the stress reduction factor based on the different thermal shock situations. Nevertheless, further experimental validation is also needed in the near future.

### 3.2. Limitations of the Applicable Range of the Second TSR Parameter

**Figure 6:**
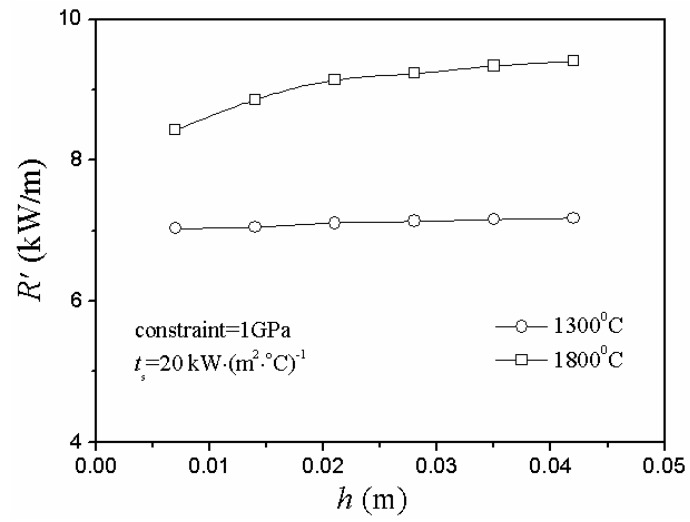
Relationship between the second thermal shock resistance (TSR) parameter, *R’*, and plate thickness, *h*, under different initial thermal shock temperature of cooling conditions.

In the case of cooling conditions, Figure 5 shows that the critical rupture temperature difference decreased gradually and slowly approached a constant as the plate thickness increased, while the second TSR parameter changed in a totally opposite way in Figure 6: as the plate thickness increased, the value of *R’* increased slightly and, finally, slowly approached a constant.

**Figure 7:**
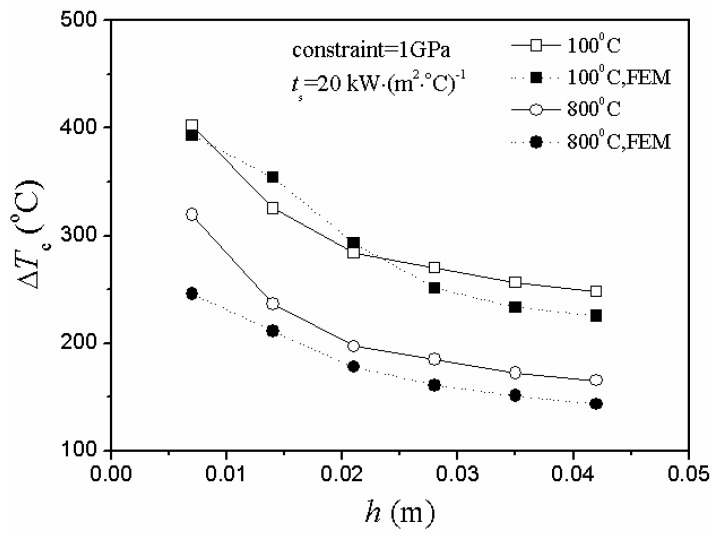
Relationship between critical rupture temperature difference, ∆*T_c_*, and plate thickness, *h*, including modified and numerical simulation situations under different initial thermal shock temperature of temperature elevated conditions.

**Figure 8:**
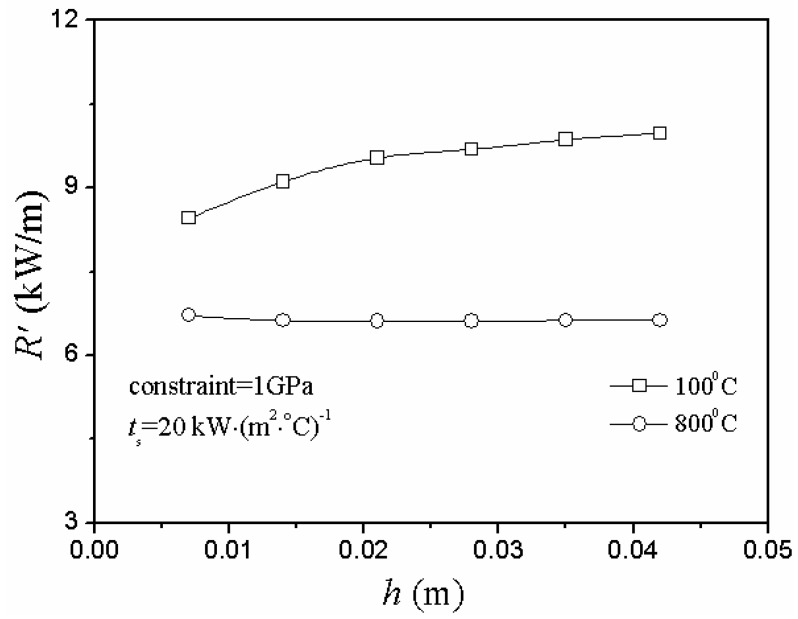
Relationship between the second TSR parameter, *R’*, and plate thickness, *h*, under different initial thermal shock temperature of temperature elevated conditions.

In the case of heating conditions, Figure 7 and Figure 8 show the same conclusion: the second TSR parameter and critical rupture temperature difference changed in totally different ways.

Besides, the theoretical results agreed well with the numerical simulation ones in Figure 4, Figure 5 and Figure 7.

Determined from the definition of the second TSR parameter, *R’* reflects the difficulty of the material damage of TSR. The greater the *R’*, the more difficult it is to initiate cracking and the better the TSR is. Moreover, *R’* can describe the actual thermal shock process better, and thus, it is superior to the first TSR parameter [18].

However, in this model, the second TSR parameter couldn’t reflect the difficulty of the material damage of TSR factually and even showed the opposite change state in either case of heating or cooling conditions. Thus, there were limitations to the applicable range of the second TSR parameter, and it was unreasonable when *R’* was used blindly to reflect all the situations of the difficulty of the material damage of TSR. Certainly, it is quite necessary to verify these conclusions by experimental research in the near future.

### 3.3. A Danger Region of Thermal Shock Initial Temperature

As it can be seen from Figure 4 and Figure 7, the 100 °C heating condition has a similar level of the critical rupture temperature difference as those for the 1,600 °C heating condition, while the 800 °C heating condition has a much lower critical rupture temperature difference.

This is because a danger thermal shock initial temperature region exists [14,17] when the critical rupture temperature difference is used to calculate the TSR of the UHTCs. Also, the phenomenon is caused by the temperature dependence of UHTC’s material properties.

The data that was calculated after the adjustment of the stress reduction factor reflect the existence of the danger region well and, thus, indicate that the thermal shock initial temperature of the ceramic plate should be as far away as possible from the danger region in the process of actual service.

## 4. Conclusions

In this paper, a temperature-dependent TSR model for UHTCs, considering the effects of the thermal environment and external constraints, was established based on the existing theory.

The adjustment of the stress reduction factor according to different thermal shock situations was considered and mainly studied in this model, and the influences of external constraint on both critical rupture temperature difference and the second TSR parameter in either case of rapid heating or cooling conditions had been studied in detail.

The results show the necessity of the adjustment of the stress reduction factor: it leads to the optimization of the theoretical values in different thermal shock situations compared with the unmodified ones; it also reflects the existence of the danger region well and, thus, indicates that the thermal shock initial temperature of the ceramic plate should be as far away as possible from the danger region in the process of actual service. There are limitations on the applicable range of the second TSR parameter, and it is unreasonable when *R’* is used blindly to reflect all the situations of the difficulty of the material damage of TSR.
